# Supplementary material for: Genome-Wide Identification and Analysis of the Hsp40/J-Protein Family Reveals Its Role in Soybean (Glycine max) Growth and Development
Source: Genes (Basel). 2023 Jun 12;14(6):1254. doi: 10.3390/genes14061254 (PMC10298129; doi:10.3390/genes14061254)
Supplement: Supplementary file 1 [file genes-14-01254-s001.zip › Supplementary Table S1.pdf]

Supplementary Table S1: The characteristics of HSP40 protein

Gene ID	Chr	Loc Start-End	Strand	CDS (bp)	PL (A.A)	PMW (kDa)	PI	GRAVY	Intron/ Exon	PD
Glyma.01G191500	01	52609448..52614224	forward	1628	560	61.900	7.32	-0.418	4/5	CHAPERONE DNAJ-DOMAIN CONTAINING PROTEIN-RELATED
Glyma.01G209800	01	54132732..54141714	forward	1625	559	59.943	9.15	-0.397	12/13	CHAPERONE PROTEIN DNAJ 13
Glyma.01G245700	01	56815425..56824795	reverse	734	251	27.099	5.37	-0.314	5/6	DNAJ HEAT SHOCK N-TERMINAL DOMAIN-CONTAINING PROTEIN
Glyma.01G036500	01	3821376..3825854	reverse	1109	422	46.255	8.79	-0.694	0/1	DNAJ HOMOLOG SUBFAMILY C MEMBER /CHAPERONE PROTEIN DNAJ 16-RELATED
Glyma.01G166000	01	50355755..50357120	reverse	479	162	17.470	9.66	-0.409	0/1	DNAJ HOMOLOG SUBFAMILY C MEMBER
Glyma.01G227100	01	55558003..55563706	reverse	2321	797	86.143	5.91	-0.569	10/11	TPR REPEAT CONTAINING PROTEIN, DNAJ HOMOLOG SUBFAMILY C MEMBER
Glyma.01G121300	01	41795454..41800252	reverse	1016	347	37.188	9.16	-0.607	2/3	HEAT SHOCK PROTEIN 40-LIKE
Glyma.01G040600	01	4389419..4392033	forward	836	285	31.487	8.48	-0.435	1/2	DNAJ HEAT SHOCK PROTEIN-RELATED
Glyma.02G013800	02	1246968..1251772	reverse	1043	356	39.122	5.60	-0.495	6/7	DnaJ homolog subfamily B member 11
Glyma.02G029600	02	2718386..2723154	forward	1244	425	46.688	8.67	-0.713	0/1	DNAJ HEAT SHOCK PROTEIN-RELATED
Glyma.02G023500	02	2120956..2123333	reverse	833	284	31.566	8.51	-0.581	1/2	DNAJ HEAT SHOCK PROTEIN-RELATED
Glyma.02G213900	02	40001857..40004507	reverse	1049	358	38.163	8.57	-0.457	0/1	CHAPERONE DNAJ-DOMAIN CONTAINING PROTEIN
Glyma.02G179900	02	30631548..30634896	forward	845	288	32.195	8.12	-1.050	7/8	DnaJ homolog subfamily C member 9
Glyma.02G211200	02	39637738..39651535	reverse	1412	483	52.588	8.90	-0.433	18/18	CHAPERONE PROTEIN DNAJ 1, MITOCHONDRIAL
Glyma.03G057500	03	8117253..8122189	reverse	1016	347	37.154	9.15	-0.646	2/3	HEAT SHOCK PROTEIN 40-LIKE
Glyma.03G242300	03	44030744..44036319	reverse	3206	1101	119.499	9.10	-0.807	0/1	DNAJ HEAT SHOCK N-TERMINAL DOMAIN-CONTAINING PROTEIN
Glyma.03G218300	03	42186853..42191345	forward	1034	353	38.805	6.29	-0.528	2/3	DnaJ homolog subfamily B member 11
Glyma.03G179800	03	39203078..39208383	forward	1475	506	54.247	5.72	-0.327	8/9	DnaJ homolog subfamily C member 3
Glyma.03G116600	03	32527884..32532444	forward	1265	432	47.148	6.65	-0.864	6/7	DNAJ HOMOLOG SUBFAMILY C MEMBER
Glyma.03G232700	03	43368821..43370172	forward	494	167	18.419	5.64	-0.894	4/5	DNAJ HOMOLOG SUBFAMILY C MEMBER
Glyma.04G094000	04	8359192..8360588	forward	743	254	27.043	5.29	-0.142	0/1	CHAPERONE DNAJ-DOMAIN CONTAINING PROTEIN
Glyma.04G196300	04	46817779..46822160	reverse	2444	839	91.780	8.71	-0.782	0/1	DNAJ HOMOLOG SUBFAMILY C MEMBER
Glyma.04G072000	04	6005853..6007339	reverse	782	267	28.750	7.79	-0.721	2/3	DNAJ HEAT SHOCK PROTEIN-RELATED
Glyma.04G175100	04	43753880..43760083	reverse	1058	364	38.914	9.04	-0.722	1/2	DNAJ HOMOLOG SUBFAMILY C MEMBER
Glyma.04G237600	04	50613748..50619304	forward	2081	714	79.128	8.78	-0.837	0/1	DNAJ HOMOLOG SUBFAMILY C MEMBER
Glyma.05G177100	05	36577540..36583451	reverse	1304	447	46.864	9.34	-0.444	6/7	MOLECULAR CHAPERONE DNAJ
Glyma.05G119200	05	31210648..31211501	forward	569	194	21.549	9.81	-0.876	0/1	DNAJ HOMOLOG SUBFAMILY C MEMBER
Glyma.05G021300	05	1882385..1885780	forward	1865	604	67.340	5.31	-1.205	2/3	DnaJ homolog subfamily A member 5
Glyma.05G152800	05	34653706..34656176	reverse	557	190	21.333	9.79	-0.742	1/2	DNAJ HOMOLOG SUBFAMILY C MEMBER

Glyma.06G289000	05	47766805..47769861	reverse	1061	362	38.454	9.05	-0.616	2/3	DNAJ HOMOLOG SUBFAMILY C MEMBER
Glyma.06G189700	06	16634347..16639910	forward	1058	361	38.698	9.03	-0.630	1/2	DNAJ HOMOLOG SUBFAMILY C MEMBER
Glyma.06G169500	06	14152798..14157127	forward	2385	819	89.197	8.73	-0.718	0/1	DNAJ HOMOLOG SUBFAMILY C MEMBER
Glyma.06G073300	06	5651330..5654202	reverse	983	336	35.898	7.73	-0.664	2/3	DnaJ superfamily
Glyma.06G126400	06	10356649..10362151	reverse	2081	714	78.840	8.16	-0.810	0/1	DNAJ HOMOLOG SUBFAMILY C MEMBER
Glyma.07G043100	07	3578232..3583129	reverse	677	230	25.023	5.15	-0.849	5/6	DNAJ HOMOLOG SUBFAMILY C MEMBER
Glyma.07G197600	07	36595253..36599804	reverse	1472	505	54.271	9.02	-0.341	3/4	DnaJ superfamily
Glyma.07G150700	07	18133085..18137499	forward	1043	356	37.595	9.29	-0.616	2/3	HEAT SHOCK PROTEIN 40-LIKE
Glyma.07G110200	07	11240291..11244896	reverse	1263	432	47.144	6.52	-0.880	6/7	DNAJ HOMOLOG SUBFAMILY C MEMBER
Glyma.07G103600	07	9945159..9951647	reverse	1580	541	57.886	8.94	-0.458	10/11	DNAJ HOMOLOG SUBFAMILY C MEMBER
Glyma.07G252300	07	42987862..42992173	forward	2879	988	108.896	6.34	-0.783	0/1	DNAJ DOMAIN-CONTAINING PROTEIN
Glyma.07G152900	07	18633135..18637055	forward	1745	598	63.721	9.29	-0.454	6/7	DNAJ DOMAIN-CONTAINING PROTEIN
Glyma.08G180300	08	14438330..14439308	forward	518	175	19.736	9.75	-0.832	0/1	DNAJ HOMOLOG SUBFAMILY C MEMBER
Glyma.08G109700	08	8419649..8422508	forward	563	192	21.592	9.73	-0.882	0/1	DNAJ HOMOLOG SUBFAMILY C MEMBER
Glyma.08G180100	08	14426404..14426920	forward	518	175	19.702	9.75	-0.809	0/1	DNAJ HOMOLOG SUBFAMILY C MEMBER
Glyma.08G024300	08	1936670..1938402	forward	548	187	20.684	8.15	-0.603	2/3	DNAJ HOMOLOG SUBFAMILY C MEMBER
Glyma.08G152100	08	11680529..11688464	forward	1337	458	48.945	8.86	-0.516	17/18	DNAJ HOMOLOG SUBFAMILY C MEMBER
Glyma.08G074200	08	5668290..5669160	forward	590	201	22.286	9.77	-0.840	0/1	DNAJ HOMOLOG SUBFAMILY C MEMBER
Glyma.08G213400	08	17225311..17230465	forward	1337	458	48.278	8.25	-0.297	10/11	MOLECULAR CHAPERONE DNAJ
Glyma.08G295500	08	41044065..41046731	forward	872	297	32.994	9.31	-0.717	1/2	DNAJ HEAT SHOCK PROTEIN-RELATED
Glyma.08G134400	08	Glyma.08G134400	reverse	1316	451	47.117	9.22	-0.425	6/7	MOLECULAR CHAPERONE DNAJ
Glyma.08G337100	08	45406335..45408065	forward	473	160	17.313	9.24	-0.559	8/9	DNAJ HOMOLOG SUBFAMILY C MEMBER
Glyma.08G238600	08	20370124..20371352	reverse	743	254	28.190	6.66	-0.350	1/2	DNAJ HOMOLOG SUBFAMILY C MEMBER
Glyma.09G044000	09	3755497..3761683	reverse	1079	368	41.088	9.24	-0.745	0/1	K09518 - DnaJ homolog subfamily B member 12
Glyma.09G075400	09	8193510..8201402	forward	2060	707	76.686	5.43	-0.260	9/10	K09540 - translocation protein SEC63 (SEC63, DNAJC23)
Glyma.09G003500	09	293204..300873	forward	1334	457	48.515	8.77	-0.431	17/18	DNAJ HOMOLOG SUBFAMILY C MEMBER
Glyma.09G283400	09	9893144..49897583	reverse	1457	500	53.608	9.06	-0.328	3/4	DnaJ superfamily
Glyma.09G155400	09	37848194..37851767	forward	2441	838	91.459	8.60	-0.846	0/1	DNAJ HOMOLOG SUBFAMILY C MEMBER
Glyma.10G094400	10	13527589..13531121	forward	848	289	32.463	8.78	-1.038	7/8	K09529 - DnaJ homolog subfamily C member 9
Glyma.10G252200	10	48006798..48010531	reverse	920	315	35.770	9.78	-0.551	9/10	K19371 - DnaJ homolog subfamily C member 25
Glyma.10G158200	10	39215031..39216459	reverse	878	299	32.305	9.34	-0.467	2/3	DnaJ homolog subfamily C
Glyma.10G272200	10	49433099..49438894	forward	1235	418	44.958	6.00	-0.558	10/11	DNAJ HOMOLOG SUBFAMILY C MEMBER

Glyma.10G158300	10	39221638..39223764	reverse	959	328	34.700	9.65	-0.559	0/1	DNAJ HEAT SHOCK N-TERMINAL DOMAIN-CONTAINING PROTEIN
Glyma.11G032400	11	2365583..2374583	reverse	1583	542	58.644	9.18	-0.410	13/14	DNAJ HOMOLOG SUBFAMILY C MEMBER 11 HOMOLOG
Glyma.11G050600	11	3779987..3786583	reverse	1628	559	61.936	7.59	-0.394	4/5	CHAPERONE DNAJ-DOMAIN CONTAINING PROTEIN-RELATED
Glyma.11G095300	11	7222139..7235534	forward	3479	1196	127.326	5.93	-0.612	10/11	TPR REPEAT CONTAINING PROTEIN , DNAJ HOMOLOG SUBFAMILY C MEMBER
Glyma.11G241000	11	33526492..33531210	forward	1325	454	47.425	9.38	-0.438	6/7	MOLECULAR CHAPERONE DNAJ
Glyma.11G077400	11	5808746..5809882	forward	479	162	17.417	9.54	-0.407	0/1	DNAJ HOMOLOG SUBFAMILY C MEMBER
Glyma.12G021400	12	1535586..1546642	forward	3458	1189	126.757	5.75	-0.585	10/11	DNAJ HOMOLOG SUBFAMILY C MEMBER
Glyma.12G239500	12	39830884..39838563	reverse	1334	457	48.619	8.91	-0.500	17/18	DNAJ HOMOLOG SUBFAMILY C MEMBER
Glyma.12G117900	12	12095818..12098847	forward	1052	359	38.238	9.06	-0.592	2/3	DNAJ HEAT SHOCK PROTEIN
Glyma.12G001000	12	77789..78816	reverse	761	260	29.001	5.93	-0.431	1/2	DNAJ HOMOLOG SUBFAMILY C MEMBER
Glyma.13G235500	13	34609106..34611350	reverse	929	318	34.182	9.16	-0.620	9/10	DNAJ HOMOLOG SUBFAMILY C MEMBER
Glyma.13G289600	13	38976042..38980726	reverse	1022	349	38137	6.14	-0.511	0/1	DNAJ HOMOLOG DNJ-5
Glyma.13G338600	13	43110251..43113828	forward	845	288	31.968	8.22	-0.935	3/4	DNAJ HOMOLOG SUBFAMILY C MEMBER
Glyma.13G214300	13	32769352..32774554	reverse	2909	1000	110.519	6.10	-0.749	0/1	DNAJ DOMAIN-CONTAINING PROTEIN
Glyma.13G036700	13	11480769..11486877	reverse	1151	394	43.618	6.35	-0.575	9/10	DNAJ HOMOLOG SUBFAMILY C MEMBER
Glyma.13G235400	13	34601528..34604996	reverse	1025	350	37.639	6.93	-0.530	2/3	DNAJ HEAT SHOCK PROTEIN
Glyma.14G010600	14	817936..818830	forward	431	160	15.954	8.78	-0.468	0/1	DNAJ HOMOLOG SUBFAMILY C MEMBER
Glyma.14G181600	14	44444730..44447403	reverse	1097	376	40.435	9.09	-0.435	7/8	CHAPERONE DNAJ-DOMAIN CONTAINING PROTEIN
Glyma.14G118300	14	15889764..15895675	reverse	1151	394	43.516	6.35	-0.575	9/10	DNAJ HOMOLOG SUBFAMILY C MEMBER
Glyma.14G178800	14	43987724..44003499	reverse	1412	450	48.686	8.75	-0.492	18/18	CHAPERONE PROTEIN DNAJ 1, MITOCHONDRIAL
Glyma.15G035700	15	2836777..2840795	reverse	863	294	32.700	6.14	-0.886	4/5	DNAJ HOMOLOG SUBFAMILY C MEMBER
Glyma.15G149100	15	12287242..12293189	forward	1088	373	41.507	9.04	-0.761	0/1	DnaJ homolog subfamily B member 12
Glyma.15G077900	15	5996629..5999931	reverse	1013	346	37.313	8.28	-0.510	9/10	DNAJ HEAT SHOCK PROTEIN
Glyma.15G006900	15	569215..577981	reverse	1484	509	53.352	8.82	-0.437	10/11	DNAJ-LIKE PROTEIN
Glyma.15G271500	15	50871306..50878174	forward	1337	458	48.967	8.85	-0.557	17/18	DNAJ HOMOLOG SUBFAMILY C MEMBER
Glyma.15G098900	15	7688067..7693423	forward	2909	1000	110.198	6.13	-0.739	0/1	DNAJ DOMAIN-CONTAINING PROTEIN
Glyma.15G183400	15	18240667..18247283	forward	2060	707	76.659	5.42	-0.242	9/10	K09540 - translocation protein SEC63 (SEC63, DNAJC23)
Glyma.15G077700	15	5986403..5988453	forward	1022	349	37.577	8.62	-0.616	2/3	DNAJ HOMOLOG SUBFAMILY C MEMBER
Glyma.16G127600	16	27950346..27950844	reverse	500	170	19.012	9.48	-0.559	10/11	DNAJ HOMOLOG SUBFAMILY C MEMBER
Glyma.16G127700	16	27974773..27975479	reverse	476	161	17.553	9.33	-0.573	0/1	DNAJ HOMOLOG SUBFAMILY C MEMBER
Glyma.16G011500	16	991886..995617	reverse	707	240	26.101	4.93	-0.917	5/6	DNAJ HOMOLOG SUBFAMILY C MEMBER
Glyma.16G205900	16	36650238..36655749	forward	2342	803	87.107	9.06	-0.776	0/1	DNAJ HOMOLOG SUBFAMILY C MEMBER

Glyma.17G078100	17	6096536..6099731	reverse	1883	646	71.576	5.10	-1.194	2/3	DnaJ homolog subfamily A member 5 (DNAJA5)
Glyma.17G022200	17	1605488..1609544	reverse	3137	990	109.108	5.77	-0.722	2/3	DNAJ HOMOLOG SUBFAMILY C MEMBER
Glyma.18G127500	18	17096118..17098853	reverse	872	297	32.888	9.30	-0.669	1/2	DNAJ HOMOLOG SUBFAMILY C MEMBER
Glyma.18G072300	18	6818984..6820432	forward	458	155	16.680	9.29	-0.572	1/2	DNAJ HOMOLOG SUBFAMILY C MEMBER
Glyma.18G201600	18	48163733..48168274	forward	1022	349	37.301	9.21	-0.653	2/3	HEAT SHOCK PROTEIN 40-LIKE
Glyma.18G016200	18	1156404..1160766	reverse	1325	454	47.565	9.27	-0.407	6/7	MOLECULAR CHAPERONE DNAJ
Glyma.18G204000	18	48677254..48680846	forward	1736	595	63.552	9.35	-0.474	2/3	DNAJ DOMAIN-CONTAINING PROTEIN
Glyma.19G239700	19	48789152..48796215	reverse	3179	1092	118.619	9.08	-0.789	0/1	DNAJ HEAT SHOCK N-TERMINAL DOMAIN-CONTAINING PROTEIN
Glyma.19G143100	19	40408473..40411444	reverse	839	286	32.011	6.87	-1.065	10/11	DnaJ homolog subfamily C member 9 (DNAJC9)
Glyma.19G215100	19	46807026..46811823	forward	1034	353	38.845	6.19	-0.522	2/3	DnaJ homolog subfamily B member 11 (DNAJB11)
Glyma.19G064700	19	16056898..16061356	reverse	551	187	20.337	9.34	-0.714	9/10	DNAJ HOMOLOG SUBFAMILY C MEMBER
Glyma.19G112100	19	36610813..36614632	reverse	926	317	34.808	7.72	-0.211	2/3	DNAJ HOMOLOG SUBFAMILY C MEMBER
Glyma.19G180600	19	43944096..43949261	forward	1511	518	55.170	5.98	-0.296	8/9	DnaJ homolog subfamily C member 3 (DNAJC3)
Glyma.20G141300	20	38004324..38007997	forward	920	315	35.708	9.38	-0.542	9/10	DnaJ homolog subfamily C member 25 (DNAJC25)
Glyma.20G230400	20	46445458..46448417	forward	2132	731	78.730	8.70	-0.728	0/1	DNAJ HEAT SHOCK N-TERMINAL DOMAIN-CONTAINING PROTEIN
Glyma.20G117900	20	36067232..36080533	reverse	1235	422	45.345	6.09	-0.584	10/11	DNAJ HOMOLOG SUBFAMILY C MEMBER
Glyma.20G002400	20	235326..239972	forward	1496	513	55.150	9.17	-0.304	4/5	Molecular chaperone (DnaJ superfamily)
Glyma.20G013600	20	1214019..1216156	Forward	527	178	19.946	5.06	-0.753	1/2	DNAJ HOMOLOG SUBFAMILY C MEMBER

Gene ID: Gene transcript ID, Chr: Chromosome number, Loc Start-End: gene start and end physical position, Strand: Gene present on forward or reverse strand, CDS (bp): Coding sequence bp, PL: Length of protein (A.A), PMW (kDa): Protein Molecular Weight, pI: isoelectric point, GRAVY: Grand average of hydropathicity, Intron/Exon: Number of introns and exons, PD: Phytozome description
